# Regulating Tradeoffs to Improve Rice Production

**DOI:** 10.3389/fpls.2017.00171

**Published:** 2017-02-09

**Authors:** Hiroshi Takatsuji

**Affiliations:** Disease Resistant Crops Research Unit, GMO Research Center, National Institute of Agrobiological SciencesTsukuba, Japan

**Keywords:** rice, tradeoff, WRKY45, WRKY62, OsNPR1, pathogen defense, stress tolerance, photosynthesis

## Abstract

Plants are sessile organisms that are continuously exposed to a wide range of environmental stresses. To cope with various stresses using limited resources, plants have evolved diverse mechanisms of “tradeoff” that enable the allocation of resources to address the most life-threatening stress. During our studies on induced disease resistance in rice, we have found some important phenomena relevant to tradeoffs between biotic and abiotic stress responses, and between stress response and plant growth. We characterized these tradeoff phenomena from viewpoints of signaling crosstalks associated with transcriptional regulation. Here, I describe following topics: (1) PTP1-dependent increased disease susceptibility of rice under low temperature and high salinity conditions, (2) OsNPR1-dependent tradeoff between pathogen defense and photosynthesis, (3) tradeoff between pathogen defense and abiotic stress tolerance in WRKY45-overexpressing rice plants, and (4) WRKY62-dependent tradeoff between pathogen defense and hypoxia tolerance. Lastly, I discuss my view regarding the significance of such tradeoffs in agricultural production that should be considered in crop breeding; that is, the tradeoffs, although they benefit plants in nature, can be rather disadvantageous in agricultural production.

## Introduction

Plants are sessile organisms that are continuously exposed to a wide range of environmental stresses (Tian et al., 2003; Matyssek et al., 2005). To cope with various stresses using limited resources, plants have evolved diverse mechanisms that enable the allocation of resources to address the most life-threatening stress. Therefore, tradeoffs exist between stress responses and plant growth or between responses to different stresses. These tradeoffs are often regulated by crosstalk between signaling pathways (Fujita et al., 2006; Pieterse et al., 2012; Sharma et al., 2013; Xiao et al., 2013; Takatsuji and Jiang, 2014). Signaling molecules such as plant hormones (Lozano-Duran et al., 2013; Huot et al., 2014; Verma et al., 2016), reactive oxygen and nitrogen species (Considine et al., 2015), and Ca^2+^ (Mazars et al., 2010) have been implicated in these crosstalks. However, the precise molecular mechanisms involved are yet to be investigated. My laboratory has been studying the salicylic acid (SA) defense signaling pathway in rice with emphasis on signaling crosstalks that mediate the tradeoffs between pathogen defense and abiotic stress responses and/or plant growth. In this review, I provide an overview of these studies, which mainly highlight a negative aspect of tradeoffs that can reduce crop production. I also propose that crop productivity could be improved by regulating tradeoffs through the inhibition of crosstalk between signaling pathways.

## Increased Disease Susceptibility Of Rice Under Low Temperature And High Salinity Conditions

**FIGURE 1:**
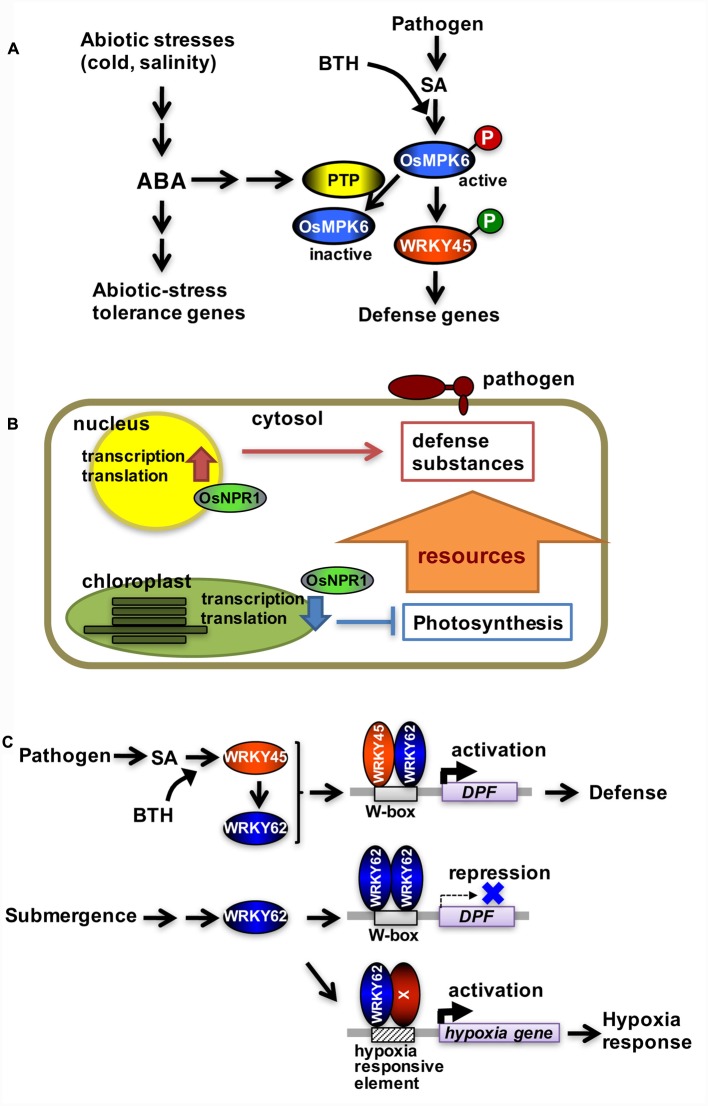
**Tradeoffs involving the salicylic acid pathway in rice. (A)** Tradeoff between pathogen defense and abiotic stress tolerance mediated by protein tyrosine phosphatase. WRKY45 is phosphorylated and activated by OsMPK6 in response to chemical defense inducers. OsMPK6 is inactivated following tyrosine dephosphorylation by protein tyrosine phosphatase, which is mediated by ABA, in response to cold stress. This leads to hypo-phosphorylation and inactivation of WRKY45. **(B)** Tradeoff between pathogen defense and photosynthesis mediated by OsNPR1. OsNPR1 downregulates chloroplastic activity resulting in a decreased photosynthetic rate, while it upregulates the expression of defense genes. **(C)** Tradeoff between pathogen defense and submergence tolerance mediated by WRKY62. Following the activation of the salicylic acid pathway, WRKY45 and WRKY62 form heterodimers that activate *DPF* transcription. Upon submergence, only WRKY62 is produced, resulting in the formation of homodimers that repress *DPF* expression. Molecule X represents a presumptive transcription factor that binds to a hypoxia-responsive element in the promoter of hypoxia-responsive genes, possibly as a heterodimer with WRKY62.

Rice blast is one of the most serious crop diseases worldwide. Chemical defense inducers affecting the SA signaling pathway have been widely used to protect rice plants from diseases such as rice blast. However, rice plants are more susceptible to blast disease when exposed to specific abiotic stresses, including low temperature, drought, and high salinity (Kahn and Libby, 1958; Bonman et al., 1988; Gill and Bonman, 1988), even in the presence of chemical defense inducers (Ueno et al., 2015). These observations seem to reflect prioritization of abiotic stress responses over blast disease resistance in rice because the abiotic stresses are often more life-threatening than blast disease. This is one of the typical tradeoffs between plant responses to abiotic and biotic stresses. By analyzing the molecular mechanism underlying this phenomenon, we demonstrated that abscisic acid (ABA) signaling, which was activated by cold and high salinity leading to abiotic stress responses, inactivated WRKY45, the central transcription factor in the SA defense signaling pathway in rice (**Figure 1A**) (Jiang et al., 2010; Yazawa et al., 2012; Ueno et al., 2015). In response to the chemical defense inducer benzothiadiazole (BTH), WRKY45 was activated by a MAP kinase cascade (OsMPKK10-2–OsMPK6)-catalyzed phosphorylation at its carboxyl terminus (Matsushita et al., 2013; Ueno et al., 2013, 2015). This led to an increase in *WRKY45* expression through WRKY45 autoregulation of its own transcription (Nakayama et al., 2013). OsMPK6 was activated by a dual phosphorylation of its TEY motif in response to dexamethasone-induced production of a constitutively active form of OsMPKK10-2 (OsMPKK10-2^D^), which mimics the activation of the SA pathway (Ueno et al., 2015). However, in the presence of ABA, OsMPK6 was dephosphorylated at its tyrosine residue even after the induction of OsMPKK10-2^D^, which decreased *WRKY45* transcript abundance and reduced blast resistance (Ueno et al., 2015).

The rice genome encodes two protein tyrosine phosphatases (PTP1 and PTP2) that dephosphorylate OsMPK6 at its tyrosine residue *in vitro* (**Figure 1A**). Knockdown of the two *PTP* genes by RNA interference in transgenic rice plants increased the abundance of TEY-phosphorylated OsMPK6 following SA treatment because of suppressed tyrosine dephosphorylation (Ueno et al., 2015). In *PTP*-knockdown rice lines, the ABA-mediated inhibition of *WRKY45* expression was considerably reduced. A blast resistance test indicated that ABA greatly suppressed BTH-induced blast resistance in untransformed (control) rice plants, whereas it did not affect the resistance of *PTP*-knockdown rice plants (Ueno et al., 2015). Low temperature (i.e., 15°C/8°C, day/night cycle) and high salinity (250 mM NaCl) conditions also suppressed BTH-induced blast resistance, but did not affect the induction of blast resistance in *PTP*-knockdown rice plants (Ueno et al., 2015). Thus, *PTP* knockdown eliminates the crosstalk between ABA and SA signaling pathways, which prevents abiotic stresses from suppressing the chemical-induced blast resistance.

One of the concerns regarding the effects of *PTP* knockdown is whether it affects normal ABA-mediated plant responses to abiotic stresses. In other words, are PTP-knockdown rice plants less tolerant to cold and/or high salinity stresses? It currently appears they are not. The induction of *SalT* expression, which is a marker gene for ABA responses, is not influenced by *PTP* knockdown (Ueno et al., 2015). Additionally, we did not observe any differences between *PTP*-knockdown and control rice plants under our low temperature and high salinity conditions. Therefore, the effects of *PTP* knockdown appear to be specific to the crosstalk between the ABA and SA pathways, and do not affect normal ABA-mediated abiotic stress tolerance.

## Tradeoff Between Pathogen Defense And Photosynthesis

OsNPR1/NH1 is an important transcriptional co-activator acting in the rice SA pathway along with WRKY45 (Sugano et al., 2010). A transcriptome analysis using *OsNPR1/NH1*-knockdown rice lines with or without BTH treatment revealed an interesting function of OsNPR1/NH1 in the tradeoff between pathogen defense and photosynthesis (**Figure 1B**). While most of the WRKY45-dependent BTH-responsive genes were upregulated by BTH (Nakayama et al., 2013), more than half of the OsNPR1/NH1-dependent BTH-responsive genes were downregulated by BTH (Sugano et al., 2010). The OsNPR1/NH1-dependent BTH-responsive genes included most of the photosynthetic genes involved in light and dark reactions (Sugano et al., 2010). The majority of genes involved in chloroplastic protein synthesis, such as the 30S and 50S ribosomal genes, also experienced OsNPR1/NH1-dependent downregulation by BTH (Sugano et al., 2010). Additionally, sigma factors involved in chloroplastic transcription were regulated in a similar manner. In contrast, genes associated with cytoplasmic protein synthesis, such as the 40S and 60S ribosomal genes, underwent OsNPR1/NH1-dependent upregulation by BTH (Sugano et al., 2010). Photosynthetic parameter measurements (i.e., Fv/Fm) in rice leaves revealed that photosynthetic activity declined more rapidly in control plants than in *OsNPR1/NH1*-knockdown plants during BTH treatments (Sugano et al., 2010). These results indicate that BTH-mediated decreases in photosynthetic activity depend on OsNPR1/NH1 (**Figure 1B**). This regulation probably represents a tradeoff prioritizing pathogen defense over chloroplastic activity unnecessary for pathogen defense. Results from a previous study (Wang et al., 2006) indicated that *Arabidopsis* NPR1 plays a similar role in the tradeoff between pathogen defense and chloroplastic activity, suggesting this regulation is common in monocots and dicots (Sugano et al., 2010).

## Tradeoff Between Pathogen Defense And Abiotic Stress Tolerance In WRKY45-Overexpressing Rice Plants

Tradeoffs between pathogen defense and tolerance to abiotic stresses were observed in *WRKY45*-overexpressing (*WRKY45*-ox) rice plants. *WRKY45* overexpression conferred rice with strong resistances to blast and leaf-blight diseases (Shimono et al., 2007, 2012). However, the growth of *WRKY45*-ox rice plants was significantly impaired compared with that of control plants, and varied with environmental conditions (Tao et al., 2011; Goto et al., 2015). Analysis of this phenomenon revealed that low temperature and high salinity conditions severely impaired the growth and viability of *WRKY45*-ox rice plants, which represents a tradeoff prioritizing pathogen defense over abiotic-stress tolerance. Following exposure to low temperatures (e.g., 8°C) for 7 days and recovery at room temperature for 7 days, 80% of *WRKY45*-ox rice plants died, while all control plants survived (Goto et al., 2015). Additionally, 75% of *WRKY45*-ox rice plants died after being irrigated with 250 mM NaCl and then water for 7 days, while 0–13% of control plants survived (Goto et al., 2015). Thus, *WRKY45*-ox rice plants were more sensitive to low temperature and high salinity conditions. This phenomenon seems to represent a tradeoff in *WRKY45*-ox rice plants, whereby the plants gained pathogen resistance through *WRKY45* overexpression, but became more susceptible to the effects of low temperature and high salinity.

## WRKY62-Dependent Tradeoff Between Pathogen Defense And Hypoxia Tolerance

Rice plants are believed to become more susceptible to diseases such as rice blast and bacterial leaf blight after submergence. This is presumably due to a tradeoff mechanism prioritizing hypoxia tolerance over disease resistance. Although the mechanism responsible for this phenomenon has not been characterized, we have identified an important WRKY62 function that is likely involved. WRKY62 is a transcriptional repressor that is regulated downstream of WRKY45. Overexpression of *WRKY62* in rice plants leads to increased susceptibility to leaf blight, which suggests WRKY62 is a negative regulator of disease resistance (Peng et al., 2008). However, our analysis of *WRKY62*-knockdown rice plants revealed that WRKY62 is a positive regulator of diterpenoid phytoalexin biosynthetic genes and other defense genes (Fukushima et al., 2016). We subsequently determined that WRKY62 can activate and repress the expression of defense genes (Fukushima et al., 2016).

WRKY62 forms a homodimer or a heterodimer with WRKY45 depending on environmental conditions (Fukushima et al., 2016). The WRKY62 homodimer functions as a transcriptional repressor of the *DPF* gene, which encodes a transcription factor that regulates diterpenoid phytoalexin biosynthetic genes (**Figure 1C**). Conversely, the WRKY45-WRKY62 heterodimer serves as a transcriptional activator of the *DPF* gene (**Figure 1C**). The *WRKY45* and *WRKY62* expression levels are similar following the activation of the SA pathway, which facilitates the formation of the heterodimer (Fukushima et al., 2016). However, under the hypoxic conditions created by submergence, only *WRKY62* is expressed, leading to the formation of the homodimer. This selective induction of transcription factor genes most likely explains why *DPF* is expressed when the SA signaling pathway is activated, but is suppressed in submerged plants. Furthermore, WRKY62 regulates hypoxia-responsive genes, including *alcohol dehydrogenase 2*, *acyl desaturase*, and *EFR*, in a manner opposite from that of *DPF.* WRKY62 functions as a positive regulator of hypoxia-responsive genes under hypoxic conditions (Fukushima et al., 2016). Thus, WRKY62 acts as a toggle switch between the expression of defense or hypoxia-responsive genes.

## Significance Of Tradeoffs In Nature And Agricultural Production

We have identified a variety of tradeoffs in rice. The tradeoffs increased susceptibility to diseases, impaired photosynthesis, or decreased tolerance to abiotic stress to prioritize responses to the most damaging stresses in specific conditions. These tradeoffs can be considered beneficial for plants because they increase survival rates, which may explain why they have developed during evolution. However, some tradeoffs may not necessarily be beneficial in agricultural production because prioritizing plant survival often results in decreased crop yields. Resource availability can differ considerably between natural and agricultural settings. Under natural conditions, available resources are often severely limited, which constrains plants to survive by prioritizing the responses to the most life-threatening stresses at the cost of other biological processes. However, in agricultural settings, more resources are usually available because they can be supplied as fertilizers. In such situations, elimination of particular tradeoff mechanisms may allow plants to cope with multiple stresses simultaneously without affecting growth or development. An example is that the elimination of tradeoffs following *PTP* knockdown prevented any increases in rice blast susceptibility under low-temperature conditions without any adverse effects on growth (Ueno et al., 2015). Unlinking hormone-regulated immunity and plant growth is also discussed in Eichmann and Schafer (2015). As mentioned above, wide variety of tradeoffs and signaling crosstalks exists in plants. Regulating such tradeoffs could be one of directions to be considered upon designing crop improvement strategies. Characterizing the mechanisms mediating various tradeoffs will be necessary to enable their regulation.

## Author Contributions

The author confirms being the sole contributor of this work and approved it for publication.

## Conflict of Interest Statement

The author declares that the research was conducted in the absence of any commercial or financial relationships that could be construed as a potential conflict of interest.
